# Emotions in the Time of COVID-19: Affections and Impacts among the Spanish Primary Care Workforce

**DOI:** 10.3390/healthcare9121723

**Published:** 2021-12-13

**Authors:** Montserrat Pulido-Fuentes, Juan Antonio Flores-Martos, Luisa Abad-González, María Victoria Navarta-Sánchez, Laura Valera-Oviedo, Carmen Cipriano-Crespo

**Affiliations:** 1 Faculty of Health Sciences, University of Castilla-La Mancha, Avenida Real Fábrica de Sedas s/n, 45600 Talavera de la Reina, Spain; Montserrat.Pulido@uclm.es; 2 Faculty of Social Sciences, University of Castilla-La Mancha, 45600 Talavera de la Reina, Spain; JuanAntonio.Flores@uclm.es; 3 Faculty of Education Sciences and Humanities, University of Castilla-La Mancha, 16071 Cuenca, Spain; Luisa.Abad@uclm.es; 4 Faculty of Medicine, Autonomous University of Madrid, 28029 Madrid, Spain; maria.navarta@uam.es; 5 Talaverana Multiple Sclerosis Association, ATAEM, 45600 Talavera de la Reina, Spain; lauravalera.psi@gmail.com

**Keywords:** qualitative study, emotions, primary care, COVID-19

## Abstract

Background: The literature review shows that most studies on the psychological impact of COVID-19 on healthcare professionals have focused on hospital staff, with few specifically addressing the primary care workforce. This study aims to explore primary care workers’ verbal accounts of the emotions they experienced. Methods: This is a qualitative study carried out between July and December 2020 in Spain. Semi-structured interviews and focus groups were conducted with primary care workers. Data were analysed through thematic content analysis. Participants were selected using purposive sampling. Results: A total of 53 primary care workers participated in the study, of whom 38 were individually interviewed, and 15 participated in three focus groups. Our analysis revealed themes in two categories: (1) from infection to affection; and (2) affected, but not patients—a discourse based on the acceptance of their experience as part of their work in primary care, creating an ideological construct or “shield” based on emotional self-management. Conclusions: Self-reflection on the emotional impact of COVID-19 is scarce. Examples of emotional affections include an obsessive focus on hygiene, the inability to establish clear boundaries between the personal and the professional spheres, and experiencing—and having to self-manage—emotional strain.

## 1. Introduction

The COVID-19 pandemic posed an unprecedented challenge for healthcare systems worldwide, and particularly for their workforces. Frontline healthcare workers have been put in an increasingly vulnerable and fragile position due to the pandemic—something that has happened before with other health emergencies, epidemics, and pandemics. Sudden public health crises are always challenging from the viewpoint of mental health. During the 2003 Severe Acute Respiratory Syndrome (SARS) epidemic outbreak, many healthcare workers reported symptoms of post-traumatic stress disorder, depression, anxiety, and fear, putting them at higher risk of developing persistent psychological anxiety [1,2]. The HIV/AIDS epidemic in the 1980s and 1990s, the 2009 H1N1 flu pandemic, the 2013–2016 Ebola outbreak, and the 2016 Zika virus outbreak are other poignant examples of this [3]. Emerging infectious diseases also have a differential impact on healthcare workers—during the recent Ebola outbreak, infection rates among medical and nursing staff were up to 10 times higher than those in the community [4].

At the beginning of the COVID-19 outbreak, different organisations suggested that the healthcare workforce would be at higher risk of contagion. Previous research on frontline healthcare workers dealing with infectious disease outbreaks has highlighted an increase in their short- and long-term mental health problems. It has been noted that, during the 2015 Middle East Respiratory Syndrome (MERS) outbreak in Korea, healthcare workers who conducted MERS-related tasks were at significantly higher risk of developing symptoms consistent with post-traumatic stress disorder (PTSD) than other workers [5].

During the present pandemic, healthcare workers have been exposed to an increased risk of infection, with Spanish healthcare professionals being one of the most affected globally [6]. As happened during previous infectious disease outbreaks, and despite knowledge gained from these, a clear lack of protective and preventive measures has left healthcare workers exposed to disease. Fear of contagion and of infecting their families, poor working conditions, and inadequate personal protective equipment (PPE), among other factors [7] contributed to an increased sense of uncertainty when conducting their work. This has caused exhaustion, with circumstances exceeding their ability to process emotions and show restraint and resilience [8]. As a result, healthcare workers have suffered increased stress, anxiety, and depression, which have affected their health and well-being [9,10,11,12]. During the early stages of the pandemic in Spain, public health decisions were dictated by the central government, mobility was severely limited, and interregional dispersion was kept to a minimum. From June 2020, however, regional differences begun to emerge—a process intensified in the last quarter of the year. With the establishment of the so-called “new normal”, inter and intraregional mobility and dispersion increased substantially. In addition, in many regions primary care human resources were depleted, with professionals being redeployed to specialised care.

The World Health Organisation has warned about the importance of avoiding infection and other occupational risks among the healthcare workforce to ensure that they can continue to meet the health needs of their populations [10]. Therefore, it is important to examine the healthcare professionals’ working conditions—and whether these have been a priority and a source of concern for the institutions concerned. Situations of crisis always impact psychological well-being—from which nobody has been safe during the COVID-19 pandemic. However, frontline healthcare workers have been disproportionately affected. In particular, carrying out tasks that clashed with their professional ethics and witnessing the unequal distribution of healthcare resources among the population added to their moral distress [9,11]. Among the most significant factors contributing to higher anxiety and depression scores among the healthcare workforce, the following have been mentioned: longer working hours; increase in the number of patients to be cared for; decreased support from peers and supervisors; inadequate logistic support; and a decrease in their perceived professional competence [13].

The healthcare workforce relies on the support of society, while also providing social support to their patients. Psychological support can reduce the negative impact of the pandemic on workers’ health and well-being [12]. New therapeutic approaches—for example, online counselling, mindfulness, or relaxation therapies, among others—seem to offer promising results [14,15], particularly when time is an issue. However, the too-rapid implementation of mental health programmes aimed at supporting healthcare professionals treating COVID-19 patients—some of the studies published describe hospital-based interventions—might give cause for concern that safety measures might have been neglected. It has been suggested that the planning and implementation of these psychological interventions might require greater attention from the relevant authorities and institutions [16]. It is also important to consider the specific needs of each individual—their different stress factors and how to best deal with the impact of the pandemic [17]. Some of the difficulties identified for implementing specific interventions [18,19] are caused by the lack of mental health training among health professionals. For this reason, it is crucial to identify the best type of interventions to mitigate the impact of the pandemic on the psychological well-being of healthcare workers [20]. Despite the limitations of the available mental health resources, it has been noted that medical and nursing staff appreciated their importance in alleviating acute mental health problems and improving their perception of their physical health [21].

In Spain, different programmes aimed at supporting health professionals in reducing work-related anxiety and improving the regulation of their emotions were implemented during the pandemic. However, the impact of these interventions has not been assessed. Neither were they based on common, agreed-upon guidelines. The interventions offered were both online and in-person, individual and group-based. While most of the team leaders and members were clinical psychologists, many interventions were rolled out with very little preparatory training or meetings. As a consequence, clinical safety measures did not receive sufficient attention [16]. Alterations caused by pandemics require the implementation of adequate alternatives. Therefore, this is an excellent opportunity to emphasise the importance of assessing and strengthening the health systems.

Billings et al. [22] conducted a systematic review and metasynthesis of 46 qualitative studies focused on the experiences and accounts of healthcare workers during pandemics and epidemics prior to COVID-19, paying particular attention to emotions and, to a lesser degree, to the COVID-19 context. By contrast, our study—besides being based on a larger sample of participants—focuses specifically on COVID-19 experiences. It is also pioneering it its focus on primary healthcare professionals—other studies are either general in their approach or focus primarily on hospital-based workers. In Spain, Palacios-Ceña et al. [23] explored the emotions of healthcare workers through 30 semi-structured interviews with hospital-based physiotherapists from the Madrid region. In China, Xu et al. [24] conducted telephone interviews with 21 primary care practitioners, identifying the barriers they experienced while conducting their professional activities and the psychological impact of these barriers.

As Alastuey [25] (p. 161) suggests, paraphrasing [26] Hochschild as well as “emotional rules” there are “emotional expression rules”—those that define what kind of emotions social actors are allowed to express in a particular social context, to what degree, and in which circumstances. Recent literature reviews revealed that most studies on the psychological impact of COVID-19 on health professionals focus on hospital staff, with few specifically addressing the primary care and social care workforce [20,22]. Therefore, our study aimed to explore the primary care workers’ accounts of their emotions and how they verbalised them. It also examined what emotions they were “allowed to feel”—analysing ideological constructs or shields such as the concept of vocation—and what emotions they could “talk about”.

## 2. Materials and Methods

### 2.1. Design

This is a qualitative study based on semi-structured interviews and focus group discussions, following an explanatory sequential design and interpretative framework [27]. The study inductively explores the personal experiences of primary care workers during the COVID-19 pandemic—an approach that allows us to register and integrate in the analysis the voices of these professionals as individuals with agency and their experiences during this period. This study is part of a larger, mixed-methods investigation [28] whose rationale is that while quantitative data provide a general picture of the research problem, a more detailed analysis in the qualitative stage provides a better, broader, and also more refined understanding of the phenomenon studied.

The study participants were primary care workers from two Spanish public healthcare services: the Castilla-La Mancha Healthcare Service (SESCAM) and the Madrid Region Healthcare Service (SERMAS). The sample included clinical and non-clinical professionals working in public primary care settings—community-based centres offering non-specialised care: family medicine, paediatrics, nursing, physiotherapy, midwifery and perinatal health, dentistry, and collection of laboratory samples.

The regions selected for the study presented similar profiles in terms of infection rates during the first surge of the COVID-19 pandemic, management of healthcare resources, healthcare access restrictions, and economic and socio-demographic factors [29,30]. Purposive sampling was used to increase diversity in participants and results. To maximise variability in the experiences collected, sampling was based on the following criteria: demographic profiles (gender and age), professional roles (practice managers, general practitioners, pediatricians), employment status (permanent, temporary, zero-hours), and years of experience (under or over 10 years of experience). We also considered whether these workers had dependent family members (Table 1). Invitations to participate in the study were sent via institutional e-mails to more than 600 healthcare professionals working in the regional healthcare system where the study was conducted (convenience sampling). Participants had to answer questions regarding their health status, job characteristics, sociodemographic profiles, and the Burnout Clinical Subtype Questionnaire (BCSQ-36). Once the survey was completed, participants were asked whether they wanted to join the qualitative research phase, either via individual interviews or focus group discussions. A total of 677 invitations were sent, of which only 37% resulted in acceptance to join the project. From this 37% (*n* = 252), only 22% (*n* = 56) agreed to participate in the qualitative phase. We also used snowball sampling, with initial participants identifying additional subjects among their contacts. Those who expressed their willingness to participate in an individual interview or focus group and fitted the target profile were sent an e-mail with information on the study’s aims, anonymisation, and personal data processing procedures. The research team responded to queries from the participants via e-mail or telephone calls.

### 2.2. Data Collection

Data collection took place between July and December 2020. Two different methods were used to collect data and obtain a broader perspective on the experience of primary care teams: (a) semi-structured, in-depth interviews [31,32] and (b) focus groups [33,34].

A total of 38 individual interviews were conducted with primary care workers, with durations of between 45–70 min each. These followed an interview guide (Table 2). Most of these interviews were face-to-face, conducted in places selected by the participants themselves. Some professionals, however, being aware of online interviews having equivalent validity to in-person ones [35], asked for the interviews to be conducted online—either because of time constraints or to avoid the risk of infection.

Many of the interviews were conducted in the Health Sciences Faculty of Talavera de la Reina, as suggested by the participants themselves.

We also organised three focus groups to gain a broader perspective on the subject studied through open discussions among participants. Fifteen primary care workers participated in these sessions. Discussions took place in comfortable classrooms in these faculties that guaranteed confidentiality, allowing participants to express their experiences freely. Focus groups were led by a research team member (moderator) with assistance from another researcher (observer). They followed a previously established guide (Table 2) and had durations of between 60–120 min each.

Different members of the research team conducted interviews and focus groups, all of them experienced in qualitative research, and were audio-recorded. A field diary was used to register contextual issues and the researchers’ observations and impressions [36]. Ten subjects withdrew from the study on the day of the interview or focus group due to different pandemic-related issues. None of the researchers involved in data collection was work-related to any of the participants.

### 2.3. Data Analysis

Both individual interviews and focus groups were transcribed verbatim. An inductive thematic content analysis was conducted to examine the participants’ experiences, identifying emerging codes, categories, and themes [37]. Each participant was allocated an alphanumeric code for data logging, the creation of categories, and as a reference for literal quotations from their verbal accounts. Each researcher analysed the data collected independently, establishing initial codes and categories. A thematic map was prepared with themes and subthemes, illustrated with relevant quotations from the participants’ accounts. The results of the individual analyses were discussed in different team meetings, where final codes, categories, and themes were agreed upon by all members of the research team.

Participants were given the opportunity to review the audio recordings and corresponding transcriptions and analyses to confirm the researchers’ interpretation of their accounts. In addition, to enhance the quality of the study we followed the Consolidated Criteria for Reporting Qualitative Research (COREQ) checklist [38].

### 2.4. Ethical Considerations

The study was approved by the relevant Ethics Committees of each region (ref. 23/2020) and the Primary Care Central Research Commission (CCI, Madrid). It was conducted according to the guidelines of the 1964 Declaration of Helsinki and the Belmont Report.

Data collected in interviews and focus groups were handled in line with current guidelines on the ethical implications of research and anonymised following current data protection laws [39]. As noted by Tolich [40], focus groups are more problematic in terms of confidentiality and ethics than semi-structured interviews. To ensure maximum confidentiality, most of the interviews and the focus group discussions were conducted in venues unrelated to the healthcare institutions and settings in which our participants conduct their professional activities.

Besides obtaining informed consent from the participants and asking them for confidentiality on the issues discussed, the focus group moderators provided in their introduction information on the study’s aims, the basic rules of participation, and the subjects to be discussed. They also explained that, due to the delicate nature of the subjects to be discussed and the open exposure of the participants during the group discussion—despite the guarantees of confidentiality offered by the research team—if any of them felt uncomfortable before the discussion started or at any point while it was being conducted they could withdraw from the study. Informed consent was obtained from all subjects involved in the study.

Data treatment complied with the General Data Protection Regulation (EU) 679/2016 of the European Parliament and of the Council of 27 April 2016, on the protection of natural persons with regard to the processing of personal data and on the free movement of such data (GDPR), and the Spanish Organic Law 3/2018, of 5 December, on personal data protection and the guarantee of digital rights (LOPDGDD). Only members of the research team could access the data collected in this study.

## 3. Results

A total of 53 primary care workers were individually interviewed (*n* = 38) or participated in focus groups (*n* = 15). The majority were healthcare professionals (78.4%), female (72.5%), with more than 10 years of work experience in primary care (70.5%). Two main categories emerged from the analysis of their accounts, describing and summarising the emotional impact of the COVID-19 pandemic on primary care teams: (1) “from infection to affection”; and (2) “affected, but not patients”—a discourse based on the acceptance of their situation as part of their work in health care and the creation of an ideological shield based on emotional self-management. Table 3 shows literal excerpts.

### 3.1. From “Infection” to “Affection”

Participants’ accounts revealed a perception of their workplace—primary care practices—as contaminated spaces. At the end of their shifts, they were extremely cautious in order not to take the virus back into their homes—the place where they could express their fears openly. This involved an array of verbalised emotions and systematic strategies that included meticulous cleaning rituals, psychology sessions with colleagues and families, and sometimes changes in their living arrangements. These concerns caused a rollercoaster of emotions and difficulties in separating their personal lives from their work. Arriving home meant leaving behind the source of contamination—their workplace, now perceived as a dangerous and forbidding place. It made them feel vulnerable and also a potential source of infection for their loved ones. The need to protect them had a tremendous emotional impact.

#### 3.1.1. Cleaning Rituals to Cope with Fear and Obsession

Primary care workers felt “dirty”, despite the improved cleaning and disinfecting protocols implemented at work. Before entering their homes, they decontaminated themselves—either voluntarily or because they were asked to—to avoid spreading the disease to their loved ones: “I was scared of bringing the infection into my home” (UM-12, general practitioner). Cleaning rituals also acted as liminal processes, helping them reduce their anxiety levels. Some of the professionals interviewed admitted feeling “psychotic, almost obsessive” (RN-16), in particular regarding cleanliness.

Fear of infecting their families was one of their greatest concerns, which often led to changes in their family routines and living arrangements. Changes took different forms: from using different rooms to workers moving to a different house or leaving their children with relatives; other families continued living together but with increased restrictions—which caused increased distress and concern.

#### 3.1.2. Confusion between Personal and Professional vs. Establishing Clear Boundaries

Our participants described their personal and professional experiences and identities as being confused during the COVID-19 pandemic. This was due to increased demands from their institutions and populations and assuming new roles and routines that took time away from their usual duties. They repeatedly mentioned their traumatic perception of “having hit rock-bottom”—that they had been pushed to the limit through lack of protection and carelessness. Distress was the most common feeling used to describe their personal experiences—it was mentioned by almost all the participants—but also anxiety, fear of contagion, and frustration. Only a minority of primary care workers admitted to having established firm boundaries—i.e., not working or being available on the phone or through WhatsApp beyond their working hours. This attitude, however, was less explicit, with most participants tending to hide it. Those who did were professionals with more extensive working experience and were explicitly less affected. Most of them declared having ignored the news and social media to preserve their mental health.

### 3.2. Affected, but Not Patients

Despite their professional roles and training in “health care”, our study revealed a lack of self-reflection among our participants regarding their own emotional state. The only references were generic—with mentions of a collective suffering from post-traumatic stress, wondering about the medium-and long-term consequences this might have. Their reflections on their personal emotions were not assessed from a clinical viewpoint. This suggests that they did not find it easy to distance themselves from the emotional impact of the pandemic on their professional roles. Despite admitting feeling affected, they could not see themselves as patients requiring clinical and psychosocial care. Thus, they only mentioned their emotions by using generic psychiatric labels and categories.

#### 3.2.1. Emotional, Not Physical, Affections

Our participants’ discourses revealed the cost and emotional strain of dealing with the pandemic. They described feeling defenceless, abandoned, and unsupported. Their narratives, however, also showed a divide between mental and physical health—the latter having been preserved better. They also revealed feelings of self-blame, self-demand, and self-responsibility. Health workers felt guilty for not doing their jobs correctly and for the ethical issues arising from certain interventions and decisions they had to make. In an attempt to rationalise and accept the situations experienced—possibly a coping mechanism—they pointed out a lack of training and preparedness. However, they also noted a situation of structural neglect and violence that had a direct impact on the workforce. Under these circumstances, the fear of contagion made them adopt a defensive stance (‘like hedgehogs’) to protect themselves. They also considered quitting their jobs.

#### 3.2.2. Ideological Shield: I Will Not Let This Affect Me

Despite suppressing their emotions, our participants admitted that the COVID-19 pandemic had increased their workload and put an immense burden on them. Those with more extensive work experience, however, pointed out that this was part of their jobs. Professional identity and vocation were thus used as defence mechanisms—as an ideological shield. A minority of our participants even declared feeling a greater professional satisfaction for having contributed during these challenging times.

#### 3.2.3. Emotional Self-Management

The most widespread strategy among healthcare workers was emotional self-management with their families and colleagues, without seeking external professional help. Many admitted that, although their workplaces offered psychological services, they seldom used them. Only one of our participants was open about having sought professional help. In some cases, participation in this study was considered a form of therapy in itself.

However, and despite their self-control and suppressing their emotions, our participants admitted sometimes feeling overwhelmed. They were also able to differentiate between the emotions they experienced and those they openly discussed during the pandemic—those that they were “allowed to feel” and “allowed to talk about”. Having devoted themselves to constantly caring for others, they had not stopped to listen to themselves—“I was not aware, we were in the middle of a storm” (Focus Group 1)—and assess the impact on their emotions.

## 4. Discussion

Considering how seldom healthcare workers self-reflected on the emotions they experienced during the COVID-19 pandemic, it is interesting to note how these were pathologised—the data collected included labels such as anxiety, psychotic state, obsessive-compulsive disorder, paranoia, or depression. It is also important to consider that the diagnosis of most of these conditions requires a temporal perspective. The noise created by the news and social media also complicated making a correct diagnosis [41]. Emotional and psychiatric symptoms and conditions whose diagnosis, according to specialists, can take years to confirm were openly discussed. The narrative built around the emotions experienced by healthcare workers has permeated their collective perception, with an excessive pathologisation or over-diagnosis of behaviours that are in fact quite “normal” in situations of crisis. This is not to imply that epidemic outbreaks—and the control measures implemented to deal with them—do not cause mental health problems, stigmatisation, and social exclusion. Indeed, these can eventually escalate to more harmful psychological responses—including adjustment disorder and depression [42]. However, the added value of our study is that it examined the personal experiences of healthcare workers while also considering the role of social constructs in how these were perceived.

The results underscore the importance of adequate organisational support and emotional self-management during stressful events, with possible future strategies including psychoeducation—with specific training to enable workers to improve their coping skills [43,44]. Limited mental health and psychosocial support systems and the lack of well-trained psychiatrists and psychologists increase the risk of common mental disorders progressing into psychopathologies In the Spanish context, the national mental health expenditure had already been [19,45] reduced prior to the pandemic [46,47]. The percentage of the health budget spent on mental health was well below the recommended figures and lower than the European average [48]. The pandemic has revealed the weakness of the systems protecting human, technological, and care resources, the insufficient funding, and the poor development of the social system The structural weaknesses of the healthcare system also include inadequate attention for emotional and mental health—often misunderstood, even among healthcare workers [49]. After the onset of the pandemic, healthcare authorities identified the importance of reinforcing the systems designed to protect the mental and emotional health of their workforce [50]. The strategies implemented, however, require careful monitoring to ensure their effectiveness.

At the onset of the COVID-19 pandemic, to cope with the challenges it posed and find relief and comfort, primary care workers turned to strengthened mutual support relationships between colleagues. However, over the following months, as the stress and tension experienced in the workplace increased, each worker had to rely on their own resources. Families were their primary source of support in a vulnerable situation, protecting them from or reducing the psychological impact of the pandemic [51,52]. At the same time, families were also an important source of concern—fear of infecting them was a factor of psychological distress and the development of mental pathologies among healthcare workers [53]. Our study also revealed the critical role of primary care—family and community healthcare services—to protect emotional health and preserve mental health [41], also among primary care workers.

Due to the infectious nature of the COVID-19 disease, most interventions planned will likely not be delivered in person but remotely. However, a recent systematic review and meta-analysis [54]—although conducted prior to the COVID-19 pandemic—suggested that individualised interventions aimed at reducing symptoms of common mental health disorders among medical staff were less effective. Moreover, remote interventions do not alleviate the primary emotional and mental health risk factors revealed during the pandemic: excessive workload, proximity to sources of COVID-19 infection, and inadequate PPE. On the contrary, among the protective factors identified are a good understanding of COVID-19, a positive work environment, and availability of adequate PPE [54]. Other protective factors against the impact of adverse events on mental health are strengthening the resilience and sense of coherence (SOC), systemic support, and adequate knowledge [20]. Recent research has suggested that during the COVID-19 pandemic, medical and nursing staff were reluctant to participate in psychological interventions or use their institutions’ well-being plans and resources [55]. This might have been because they felt strong enough to continue working without them or because they had other priorities [56]. For instance, resting or improving the safety of their workplace. Other studies have also suggested that in-person psychological interventions are more likely to be accepted than online ones [16].

Workplace-related issues can be both risk and protective factors for mental health [20]. The number of cases in an area is also a stress risk factor. A higher incidence rate is directly associated with higher stress among healthcare workers [14]. The regions included in our study were among those with higher COVID-19 incidence rates in Spain [30]. Asymptomatic cases have also been a source of concern for primary care workers, unlike for the rest of the population [57]. It is also important to consider the protective effect of seniority—experience and self-confidence can help minimise the stress experienced in unexpected circumstances. At the same time, those healthcare workers who felt they needed psychological support but did not have time to seek it suffered from higher stress levels [53].

Despite the concept of integral health having been established for decades, our participants perceived a difference between their physical and mental health. The existing care delivery model, focused on monitoring health and disease by prioritising a biomedical approach and neglecting the social dimensions of health, might be in part a consequence of its workers’ segmented perception. This is at odds with the more widespread, all-encompassing definition of health, which considers that only the balance between physical, biological, emotional, mental, spiritual, and social aspects can sustain adequate growth and development [58,59]. A similar viewpoint underpins the concept of “One Health” [60]. On the other hand, healthcare workers’ professional identity is closely intertwined with, and predominantly values, the ideological concept of vocation. References to vocational issues frequently appeared in our participants’ narratives [61]. The importance of this viewpoint was magnified during the COVID-19 pandemic—when increased workloads pushed burdened healthcare workers to the limit of their physical and mental strength. To avoid further occupational distress, it is important to examine carefully the symbolic and structural factors underpinning the emotional effects that healthcare workers feel allowed to express when conducting their professional activities.

The challenge posed by the pandemic has also highlighted how their personal and professional identities are sometimes blurred. This duality has been noted previously in situations of conflict between health personnel and patients [62] with healthcare workers blocking and not allowing themselves to feel vulnerable. It is important to be aware of the cognitive, emotional, and even institutional barriers that need to be navigated to enable healthcare workers to seek the available psychological intervention resources they might need—and to enable them to acknowledge their psychological problems instead of hiding or obviating them. On a therapeutic level, psychological interventions in the context of the COVID-19 pandemic require a combined implementation of early intervention, monitoring, and recovery programs to help overcome stressful and traumatic symptoms [19]. Our study revealed that healthcare workers were aware of the emotions they experienced (fear, irritability, anxiety, sadness). However, they experienced difficulties in expressing those emotions without breaking down and acknowledging their limitations to self-regulate those emotions. At the same time, they demonstrated more skills and emotional intelligence when supporting their colleagues or families—helping regulate other people’s emotions. This might suggest that there was also an element of mental health stigma at play among healthcare workers in addition to the risk factors already mentioned. This is indicative of prevailing stereotypes associated with mental pathologies [63,64]. Previous studies examining emotional affections among healthcare workers [55] suggested the importance of identifying those factors that could be modified and improving training on how to prevent and address emotional problems. The issues identified in existing interventions underline the importance of an efficient combination of online tools and resources and in-person consultations, and the cooperation between different professional categories to address mental and emotional health.

The main limitations of the study are related to the care services provided at the primary care centres. Not only were these not interrupted during the pandemic, but the attention to non-COVID patients was maintained despite staffing issues due to high levels of COVID-19 infections. At the same time, this study was conducted in parallel to other research projects, which might have caused a certain degree of saturation and reluctance to participate among healthcare professionals.

Finally, it is entirely possible that those professionals who chose to participate in our study might have felt more acutely the strain of the pandemic, causing us to overestimate the severity of the situation. However, as far as we know, this is the first study that has explored the impact of the COVID-19 pandemic on primary healthcare workers [28]. This subject can be explored further in future studies—for instance, whether there are better strategies to explore pandemic-related mental and emotional health issues among healthcare professionals, particularly in primary care.

On the other hand, healthcare authorities need to be aware of rising mental health issues among their workforce, since this is not just a problem of staff wellbeing—as others have suggested, it can have a direct effect on patient safety [65].

## 5. Conclusions

During the COVID-19 pandemic, healthcare workers perceived their workplaces as risky, dirty, contaminated spaces. The transition from work to personal spaces involved obsessive cleaning rituals and implementing changes in family routines and structures, which in most cases blurred the lines between work and personal lives. Those workers who established clear boundaries were not forthcoming in admitting this publicly. Primary care workers’ perception of their health established a divide between their physical health, which was preserved, and their emotional and mental health, often affected. However, emotional affections were seldom expressed, and when they did, they were hidden under a biomedical language and the pathologisation of their symptoms. This allowed workers to create an ideological shield to self-manage their emotional and mental health, with very few seeking external help. Therefore, it is essential to implement policies to reinforce healthcare systems, particularly primary care, in order to address mental and emotional health in healthcare workers.

## Figures and Tables

**Table 1:** Profiles and characteristics of the participants in the study.

	Number of Professionals	Under or over 10 Years of Experience	Male/Female	With/Without Dependent Family Members	Rural/Urban Environment
Nurses	26 ^1^	22/4	7/19	17/9	9/17
General Practitioners	2	1/1	1/1	1/1	0/2
Nursing Managers	5	3/2	1/4	2/3	2/3
Practice Managers	4	3/1	1/2	1/3	2/2
Nursing Aides	3	2/1	0/3	2/1	1/2
Emergency Technicians	2	1/1	0/2	1/1	1/1
Social Workers	3	2/1	0/2	1/2	0/3
Physiotherapists	3	2/1	0/3	2/1	2/1
Administrative Staff	2	2/0	0/2	1/1	0/2
Midwives	2	2/0	0/2	1/1	1/1
Cleaners	1	1/0	0/1	1/0	0/1

^1^ Focus group discussions only included nursing professionals.

**Table 2:** Interview guide.

Subject Areas	Questions
Working in a primary care setting	Describe an average working day before and during the COVID-19 pandemic. How have the work management practices and care provision planning been affected? What has been the impact of the present health crisis on yourself and your family? How have you looked after yourself and your family? What have been your experiences regarding the protection and safety measures against COVID-19? How would you describe your emotional state over the past months? HWhat factors have helped you cope with your day-to-day routines since the start of the health crisis? Did you consider the risk of being infected while at work?
Working as part of a team	Describe how the relationship with the rest of the team has been. Has anything changed during this time?
Healthcare provision	How has the health crisis affected users, patients, families, the community, and primary care in general, compared to the situation before the pandemic?
	Would you like to add something else?

**Table 3:** Literal excerpts from the interviews and focus groups.

Theme 3.1 From “Infection” to “Affection”	
Category 3.1.1. Cleaning rituals to cope with fear and obsession	*I would leave with a feeling of being dirty, dirty with the virus... I felt dirty, but not with physical dirt*—*I made sure I did a really thorough cleansing, it was like another part of my* job (UN-11, nurse). *Ah, yes, it was a drama, arriving home—and my boyfriend was like, ‘Don’t touch me... Have a shower, like, wash your hair’, and you are like, I am going to turn bald. And then clean the door knobs, the food, the shopping, wash your clothes at 60 degrees* (UM-28, general practitioner). *I moved out to be alone, I haven’t seen my mother for... I’m trying to explain to her: ‘Mother, I cannot hug you, I cannot kiss you’*—*without seeing my daughters and my grandson*—*I have a four-year-old grandson, he is gorgeous. Well, it has been ages. And that, too, makes you...* (Focus Group 3).
Category 3.1.2. Confusion between personal and professional vs. establishing clear boundaries	*We had to provide our personal phone numbers, so we could be sent people’s pictures to assess them* (RN-22, nurse) *I was working almost 12 h every day, or 13 (...) No time left. I took the computer, and the telephone from the practice because it has a mobile line, I took it home so I could phone and follow up with the patients... I would switch the computer off at midnight because there was not enough time, it was constant. And emotionally, well, it was hard, I could not sleep, I lost my appetite, the situation overwhelmed us*—*the emotional cost has been tremendous, I found it really difficult* (UM-37, general practitioner). *I have become as involved as I had to, which was a lot, within the limits of the safety guidelines that we had*—*In that sense, I have not felt psychologically affected… perhaps because I have my tools… to control this kind of stress a bit*—*perhaps things that would have annoyed you a bit in the past now you would take no notice*—*situations that were not that important*—*when the clock strikes three, you leave it behind* (UN-2, nursing manager)
Theme 3.2. Affected, but not patients	
Category 3. 2.1 Emotional, not physical, affections	*My emotional state has been bad, a lot of anxiety, a feeling of becoming obsessive*—*I would wake up obsessed that I was getting infected. And then that psychotic, obsessive-compulsive state at work, assuming these incredible cleaning roles* (RN-16 nurse). *Physically I haven’t had any problem, but mentally it has been hard, really hard, a lot of fear, dread, anxiety, a lot of anxiety, and worrying about the same thing all day* (RTE- 3, healthcare technician). *I have felt anxious myself, in fact once I was in my car, driving out of the garage, and I felt unable to go on. I had to phone my husband then: ‘I can’t get out of the garage, I can’t, I am going to crash...’ And he was like, ‘But stay, stay, and I went, ‘No, no, I can’t stay, there is somebody and, if I do not get there... it is 1.30 and I have to be there at 2 o’clock, imagine if I let her down, she’ll be getting a phone call to get there now’. And then, I was like, ‘No, it’s ok, it’s passed now’. It was just a moment and I got there, and that was that, it was easy* (UF-31, physiotherapist). *We are not mentally or emotionally prepared, we were not prepared for this, and this is something that will leave a mark and it will be difficult to move forward*—*we have been on the front line*—*we have been overloaded at work and mentally, and it is unbearable* (UM-27, general practitioner). *As a collective, I think we adopted a stance like hedgehogs, we put our spines on and did not want to see anybody in-person until we had the resources* (URF-1, physiotherapist). *For me, it was awful. To the point that I considered quitting my job*—*even though I have been doing it for a long time. I felt that the way some centres and practices were organised put me at risk* (UN-15, nurse).
Category 3. 2.2. Ideological shield: I will not let this affect me	*On a psychological level... I had no problem at that level. I mean, this is what we have to do, this is what we have been studying for, this is what we have prepared for* (UN-14, nurse). *We are healthcare workers and that’s it, I think as a society we have turned rather soft, it was hard, for sure, it was a pandemic... So*, *let’s work* (Focus Group 1). *I think we did what we usually do, this is a vocational profession, and we give our best without considering the consequences. This ‘best healthcare system in the world’, as some were calling it, is a system that relies in the courage and dedication of its workers*—*without sufficient support or resources* (UM-26, general practitioner). *It’s made me feel professionally fulfilled*—*being able to help in a situation like this, being there during such a critical situation* (UN-13, nurse).
Category 3. 2.3. Emotional self-management	*I have managed it at home, with my husband, on my own, with my own resources, with my colleagues*—*we would talk, and cry, and get everything out of our systems, and then we would go home at ease* (UN-15, nurse). *The management provided*—*and the social worker at my practice*—*there were psychological support groups if you needed them, although I haven’t really needed them, and I don’t think any of my colleagues has used them*—*but we were given the opportunity, if we had the psychologists’ phone number, we could*—*with an email or a phone call, we could get in touch* (UF-31, physiotherapist). *Until I got my holiday break I was taking sleeping pills. But still, I came in the mornings, I would leave in the evenings exhausted, and then I would recover and so*—*to be honest I haven’t been on leave at any time (...). And it’s like I have become used to it* (UD-32, administrative staff). *Perhaps just talking to you will be like therapy, because I really cannot take any more*—*I do not use any because I don’t*—*confidentiality and all that* (RM-21, general practitioner). *I thought I was doing better, until one day something made me snap and cry, that day I cried so much... And I was like, I am not really crying because my friend’s mum has died*—*although I really knew her and all that*—*I think I am letting go of all the accumulated tension... That scared me. I was like, this is not right, this is too much*—*in fact, afterwards I had an episode of stress... My family noticed it too* (Focus Group 2).

## Data Availability

The data presented in this study are available on request from the corresponding author. The data are not publicly available due to confidentiality agreements with participants.
